# Editorial: Plant Viruses, Volume I: Detection Methods, Genetic Diversity, and Evolution

**DOI:** 10.3389/fmicb.2021.793071

**Published:** 2021-12-06

**Authors:** Akhtar Ali, Rajarshi Kumar Gaur, Xifeng Wang, Xiaofei Cheng, Kristiina Mäkinen

**Affiliations:** ^1^Department of Biological Sceince, The University of Tulsa, Tulsa, OK, United States; ^2^Department of Biotechnology, Deen Dayal Upadhyay Gorakhpur University, Gorakhpur, India; ^3^State Key Laboratory for Biology of Plant Diseases and Insect Pests, Institute of Plant Protection (CAAS), Beijing, China; ^4^Key Laboratory of Germplasm Enhancement, Physiology and Ecology of Food Crops in Cold Region of Chinese Education Ministry, College of Agriculture, Northeast Agricultural University, Harbin, China; ^5^Department of Microbiology, Viikki Plant Science Centre, University of Helsinki, Helsinki, Finland

**Keywords:** high throughput sequences (HTS), novel viruses, diversity, recombination, complete genome sequences

Both RNA and DNA plant viruses commonly infect agricultural crops worldwide and cause tremendous losses in crop yield. Infections are also common in non-cultivated host plants and trees. Over the last 40 years, the number of plant viruses identified worldwide from various plants has increased tremendously because of the availability of new diagnostic techniques.

Plant viruses are constantly emerging and new viruses are being identified at an accelerating rate worldwide. One of the main reasons for the emergence of new viruses is their constant evolution, which allows them to re-emerge as a new strain of the same virus or as a new virus to infect new hosts. Therefore, control measures for a specific plant virus will work for a short time but may not be effective for longer periods. Despite large numbers of evolutionary studies of plant viruses over the last 20 years, plant virologists are still in the early stages of understanding how these viruses evolve, interact with their hosts, and survive naturally in the wild.

In this Research Topic, we received 11 research articles, which cover topics about the identification and evolutionary analysis of new plant viruses from a variety of cultivated crops including wheat, barley, maize, pea, pepper, tomato, soybean, garlic, and Zanthoxylum armatum (a spiny shrub).

The availability of high throughput sequences (HTS) technique has made identification of plant viruses regarding their genome from both domesticated and wild plants much faster and easier than previously described methods. A recent study by Kondo et al. was focused on yellow mosaic disease (YMD), which infects winter wheat in Asian countries, specifically Japan. YMD is usually attributed to the infection of bymovirus or furovirus. In their work, they identified a new virus associated with YMD of winter wheat in Hokkaido, Japan. This new virus was named wheat virus Q (WVQ), which belongs to the subfamily *Quinvirinae* (family *Betaflexiviridae*). WVQ is potentially transmitted through soil to wheat and rye plants. The virus infection also induces antiviral RNA silencing responses in the host.

Wheat is commonly infected by wheat streak mosaic (WSM) disease complex that contains three viruses: wheat streak mosaic virus (WSMV), triticum mosaic virus (TriMV), and high plains wheat mosaic emaravirus (HPWMoV), which are all transmitted by wheat curl mites. In our Research Topic, Redila et al. presented the genetic variation based on the complete genome sequences among the isolates of three viruses associated with WSM, which were collected from wheat fields located in Kansas or Great Plains and analyzed by HTS. The results suggest the association of all three viruses with WSM disease and interestingly low genetic diversity in the natural populations of WSMV and TriMV.

Yellow dwarf viruses (YDVs; ssRNA viruses in the family *Luteoviridae*) are the most widespread group of cereal viruses worldwide and are transmitted by more than 25 aphid species in a persistent non-propagative manner. A study on barley/cereal yellow dwarf viruses using HTS by Somera et al. describes the complete genome sequences for 22 isolates of CYDV-RPS, BYDV, GAV, BYDV-PAS, BYDV-PAV, and BYDV-OYV from symptomatic plants collected from 47 cereal (wheat, barley, oat, rye, and triticale) fields in Estonia and meadow fescue in Sweden. In addition, they assembled a nearly complete genome of a novel species of BYDV-OYV from Swedish samples. Recombination analysis of the virus sequences revealed that BYDV-OYV is the parental virus for BYDV-PAV and BYDV-PAS, which further improved our knowledge about the complexity of viruses associated with YDVs.

Maize and sorghum are staple food crops and are infected by diverse pathogens to reduce their marketable yield. Maize strip virus (MSpV, Tenuivirus) infects maize, but the complete genome sequence has not been available so far. The HTS work presented by Bolus et al. reports the first complete reference genome sequence of MSpV and those of additional virus isolates collected from maize in the United States and Papua New Guinea. From these sequences, they determined genetic diversity.

Another metagenomics study on green peas (*Pisum sativum*) virome in Germany using rRNA-depleted total RNA extracts from pooled plant tissue in combination with HTS revealed 35 viruses and 9 associated nucleic acids (Gaafar et al.). Bioinformatics analysis of virus sequences and further confirmation by reverse transcription-polymerase chain reaction (RT-PCR) showed that 25 viruses are either novel strains or viruses. The most abundant viruses in peas were pea enation mottling virus 1 (PEMV1) and pea enation mottling virus 2 (PEMV2), followed by turnip yellow virus (TuYV). In addition, a number of viruses were reported for the first time to infect peas in Germany.

Soybeans are valuable cash money-making crops that provide protein rich food for both humans and animals worldwide. Soybean mosaic virus (SMV, genus *Potyvirus*) and cowpea mild mottle virus (CPMMV, genus *Carlavirus*) are the two important ssRNA viruses that infect soybean worldwide. Wei et al. reports the presence of SMV and CPMMV in soybean samples from different regions of China. The HTS results showed that both viruses co-infect soybeans. Phylogenetic analysis of 11 nearly complete genome sequences of CPMMV isolates formed a distinct clade from previously reported isolates from around the world, indicating the presence of a new genetic clade.

Cao et al. describes a new virus associated with flower yellowing disease (FYD) of *Zanthoxylum armatus*, which has been a main constraint on the production for the last 10 years in China. *Z. armatus* belongs to the family *Rutaceae*, which comprises deciduous, spiny shrub species and are cultivated for spices, medicine, and essential oils. It is also known as green Sichuan (Szechwan) pepper. *Z. armatus* is economically important crop and is cultivated for green fruits that can be used as a seasoning for its special aroma and numbing flavor. The results of HTS from different diseased trees showed the association of a new ilarvirus (family *Bromoviridae*) named green Sichuan pepper-nepovirus (GSPneV) and the satellite RNA (satGSPNeV). This work showed for the first time that FYD is caused by a virus/subviral infection.

Pepper and tomatoes are infected by a range of plant viruses and cause significant effects on the plant itself and fruit. In this Research Topic, we had three studies, which showed the identification, biological and genetic characterization of viruses infecting peppers and tomatoes. One study showed the complete genome sequence of a new recombinant polerovirus: pod pepper vein yellows virus (PoPeVYV) (Peng et al.) that was determined by HTS and further confirmed by RT-PCR. Another study (Li et al.) based on serological and RT-PCR techniques found 19 different viruses in both crops, while seven viruses were common between peppers and tomatoes. The highest infection (20%) was recorded for an orthotosposvirus and tomato spotted wilt virus (TSWV), followed by a polerovirus, pepper vein yellows virus (PeVYV, 13.0%). The results showed for the first time that pepper is a natural host of tobacco vein distorting virus (TVDV; genus *Polerovirus*) worldwide and tomato is a natural host of potato leafroll virus (PLRV; genus *Polerovirus*) in China. The third study (Rao et al.) reported the identification of two new isolates of chili veinal mottle virus (ChiVMV; family *Potyviridae*, genus *Potyvirus*). Phylogenetic analysis showed virus isolates clustered based on the geographical origin while recombination was a major factor affecting the diversity of ChiVMV isolates.

Allium spp (onion, green onion, garlic, leek) is a hub of viruses, particularly tospovirues, which is a major constraint for their production worldwide. Tabassum et al. presented a global evolutionary analysis of many iris yellow spot virus isolates (IYSV;genus *Tospovirus*, family *Bunyaviridae*) based on the complete N gene sequences (considered as a marker gene for tospovirus identification and classification). Phylogenetic and in *silico* restriction fragment length polymorphism (RFLP) analysis of the N gene of 142 IYSV isolates categorized IYSV isolates into two major gentoypes: IYSV Netherlands (IYSVNL; 55.63%), IYSV Brazil (IYSVBR; 38.73%) and the rest fell in neither group [IYSV other (IYSVother; 5.63%)]. These results suggest that IYSVNL is the predominant genotype on a global scale.

In conclusion, these articles provided novel studies and knowledge about viruses infecting cereals, peppers, tomatoes, peas, and trees. Most importantly, the identification of new viruses from various plant species or the availability of complete genome sequences of known viruses will further close the knowledge gap about these viruses. This knowledge can be applied in future studies for the identification of novel viruses and their characteristics to understand how we can protect our agricultural crops to ensure a sustainable food supply in the coming years.

## Author Contributions

AA initiated this Research Topic and invited other guest editors. AA wrote the original draft of the editorial and all other authors reviewed and approved it for publication. All authors listed above have made direct and intellectual contribution to the above topic.

## Conflict of Interest

The authors declare that the research was conducted in the absence of any commercial or financial relationships that could be construed as a potential conflict of interest.

## Publisher's Note

All claims expressed in this article are solely those of the authors and do not necessarily represent those of their affiliated organizations, or those of the publisher, the editors and the reviewers. Any product that may be evaluated in this article, or claim that may be made by its manufacturer, is not guaranteed or endorsed by the publisher.

